# Differences in Gut Microbiome Profile between Healthy Children and Children with Inflammatory Bowel Disease and/or Autoimmune Liver Disease: A Case-Control Study

**DOI:** 10.3390/pathogens12040585

**Published:** 2023-04-12

**Authors:** Robert N. Lopez, Steven T. Leach, Nerissa Bowcock, Elise Coker, Amanda J. Shapiro, Andrew S. Day, Daniel A. Lemberg

**Affiliations:** 1 Department of Gastroenterology, Sydney Children’s Hospital, Sydney 2031, Australia; 2 Department of Paediatrics, School of Clinical Medicine, University of NSW, Sydney 2052, Australia; 3 Department of Paediatrics, University of Otago Christchurch, Christchurch 8041, New Zealand

**Keywords:** autoimmune liver disease, primary sclerosing cholangitis, inflammatory bowel disease, microbiome, paediatrics

## Abstract

Background: The role of gastrointestinal microbiome in health and disease is increasingly appreciated. A significant amount of evidence clearly points to a dysbiosis manifest in inflammatory bowel disease (IBD) when compared to healthy controls. Less understood is the microbiome profile in autoimmune liver disease (AILD). Both adult and paediatric data indicate a distinct microbial signature in patients with IBD and co-existent primary sclerosing cholangitis (PSC), which is unique and different compared to the microbial signature that exists in patients with IBD alone. However, there is limited information on the microbiome make-up of patients with parenchymal liver disease, with or without IBD. Methods: The present study sought to compare the microbiome of children with IBD, to those with IBD-AILD, those with AILD alone and those of healthy controls. Results: Results from this work indicate that children with AILD have a microbiome profile that mirrors healthy controls. Conclusion: Those with IBD-AILD and IBD have similar microbiome profiles which are distinct from AILD alone and healthy controls. This suggests that the dysbiosis in these groups is primarily due to IBD rather than AILD.

## 1. Introduction

Autoimmune liver disease (AILD) can be classified as autoimmune hepatitis (AIH), primary sclerosing cholangitis (PSC) or AIH-PSC overlap syndrome, otherwise known as autoimmune sclerosing cholangitis (ASC) [1]. AIH is a relapsing and remitting condition affecting the hepatic parenchyma and serologically manifests by the derangement of liver biochemistry and the elevation of serum immunoglobulin G (and/or globulin) and the presence of specific autoantibodies. AIH is histologically characterized by, among other features, the presence of interface hepatitis. PSC, on the other hand, is a progressive, autoimmune condition, which affects the intra-hepatic and/or extra-hepatic biliary tree and is manifest by cholestatic biochemical derangement and at least one stereotypical radiological or histological change. ASC, commonly referred to simply as overlap syndrome, is a condition manifest by hepatobiliary inflammation, as individually seen in AIH and PSC—often to a less severe degree [1].

AILD can be present with or without concomitant inflammatory bowel disease (IBD) [1]. The subtypes of AILD have an individual bearing on the likelihood of co-existent IBD, with PSC having the strongest association with IBD. Furthermore, the onset of AILD can precede that of luminal disease. Of concern, the presence of sclerosing cholangitis, in the context of PSC or overlap, with IBD is associated with increased risk of colorectal carcinoma and also cholangiocarcinoma. Furthermore, the natural history of PSC diagnosed in childhood is known to be associated with adverse outcomes associated with chronic liver disease [2,3].

Changes in the intestinal microbiome have been increasingly recognised as relevant to the pathogenesis and outcomes of IBD [4,5,6]. A few recent studies have demonstrated a distinct microbiome profile in patients with PSC, with or without IBD [7,8,9]. The implications of these observations upon the outcomes of PSC (including the development of long-term complications) have not been well characterised. Furthermore, little is known about the profile of the microbiome in children with AILD, other than PSC [10]. This study sought to delineate the microbiome profile in children with AILD, with and without concomitant IBD.

## 2. Patients and Methods

### 2.1. Study Population

Ethical consent for the study was provided by Sydney Children’s Hospitals (SCH) Network Human Research Ethics Committee’s Executive Committee (LNR/16/SCHN/451). Parents and caregivers of potential participants cared for within the paediatric gastroenterology department at SCH Randwick were approached and provided with information regarding the study. Participants were included in the study following the completion of informed consent processes by their parents or guardians, and assent by the participants themselves.

Four separate groups of children were identified and recruited. Children with isolated AILD (of any type) were identified from consultant patient lists. The diagnosis of AILD was made based on a combination of liver biochemical derangement, elevated serum antibody levels (anti-nuclear antibody (ANA); liver-kidney microsomal antibody (LKM) and/or smooth muscle antibody (SMA)), elevated serum Immunoglobulin (Ig)G, stereotypical histological findings of AIH+/− PSC and/or stereotypical magnetic resonance imaging of the hepatobiliary system [1]. Children managed as AILD who did not fit these diagnostic criteria were not included.

Children with IBD with or without AILD were identified from an existing IBD Clinic database and grouped as IBD or IBD-AILD. The diagnosis of IBD was based on the ESPGHAN revised Porto criteria [11]. Finally, a group of healthy children were recruited as healthy controls (HC) from friends and family of SCH Staff. The HC group comprised well children without an existing diagnosis of gastrointestinal (GI) or liver disease, and with no current GI symptoms. Exposure to antibiotics, probiotics or prebiotics in the four weeks prior to study enrolment was an exclusion criterion for all groups.

### 2.2. Stool Sample and Data Collection

Participants were asked to provide a single stool sample using sterile collection containers (Techno-Plas, St Marys, Australia) at home. Samples were immediately placed in the home freezer at –20 °C and then transported frozen to the research laboratory where they were stored at −80 °C until analysis.

Age and sex data was collated for all children. Clinical, disease phenotypic and pertinent investigation findings of the children included in the three disease groups were obtained by perusal of their hard-copy and electronic patient records. Where there was ambiguity within the patient notes, clarification was sought from the child’s treating gastroenterologist and/or the patient and their parent/legal guardian.

### 2.3. Microbial DNA Extraction and 16S rRNA Sequencing

Faecal microbial DNA was extracted from stool samples using the Mo-Bio PowerFecal DNA Isolation Kit (Qiagen, Hilden, Germany), following the manufacturer’s protocols. DNA concentration and quality were evaluated using a Thermo Scientific NanoDrop-2000 spectrophotometer (ThermoFisher, Scoresby, Australia); DNA concentrations of 5–15 ng/µL were acceptable for sequencing. Variable region 3 and 4 (V3–V4) of the 16S rRNA encoding genes were targeted and amplified by PCR using forward primer 341F and reverse primer 805R. The resulting amplicons were sequenced on the Illumina MiSeq platform (Ilumina Inc., San Diego, CA, USA) at the Ramaciotti Centre for Genomics, University of New South Wales, Sydney, Australia.

### 2.4. Generation of Microbial Community Composition Profiles

Raw 16S rRNA sequences were processed and assembled into amplicon sequences variants (ASVs) in Rstudio version 1.4.1717 (Rstudio Inc., Boston, MA, USA) using a dada2 method workflow adapted from previously established pipelines [12]. Poor quality, chimeric and unclassified sequences at the phylum level were removed, and microbial taxonomy was assigned to the ASVs down to species classification where identifiable. Output data tables were generated for all taxonomic levels from phylum to species. Data were converted from raw abundance to relative abundance for analysis. Further analysis of alpha diversity (Observed OTUs, Simpson, Shannon and Chao1 index) and beta diversity (Bray-Curtis dissimilarities) were performed using the phyloseq pipeline and graphed via the ggplot2 package.

### 2.5. Statistical Analysis

Statistical analysis was carried out using GraphPad Prism version 7.00 for Windows, GraphPad Software, La Jolla, CA, USA, “www.graphpad.com”. Continuous variables are presented as mean with standard deviation (SD) for normally distributed data unless stated otherwise. Categorical variables are presented as frequencies (percentages). One-way ANOVA with Tukey’s multiple comparisons test were used to test for differences in α-diversity between groups. Beta-diversity was assessed by Principal component analysis. *p* < 0.05 was considered statistically significant.

## 3. Results

### 3.1. Study Population

Forty participants were included in the study. This number comprised seven with AILD alone with no IBD (AILD), six with AILD and IBD (AILD-IBD), twenty-two with IBD alone (IBD) and five without liver or intestinal disease (HC) (Table 1). Of the AILD group, four had AIH type 1, two had AIH-PSC and one had seronegative AIH. Age, gender, IBD type and the history of medications received for each group were noted (Table 1).

### 3.2. Microbiome Analysis—Alpha Diversity

**Figure 1:**
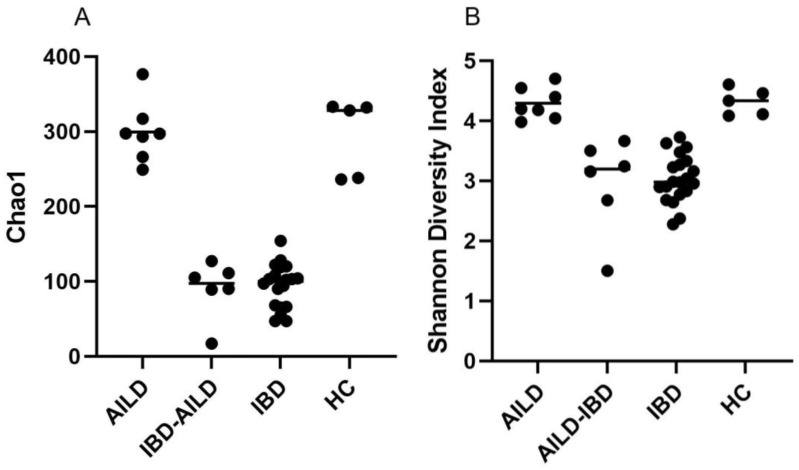
Alpha diversity. Alpha diversity of the intestinal microbiome was assessed by Chao1 (**A**) and Shannon’s Diversity index (**B**). AILD (Autoimmune Liver Disease); IBD (Inflammatory Bowel Disease); HC (Healthy Control).

**Figure 2:**
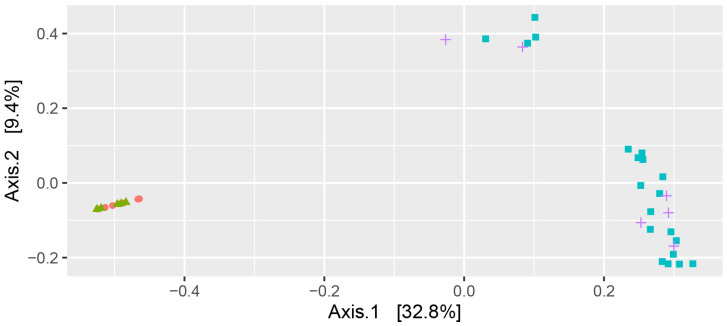
Beta Diversity. Beta diversity of the intestinal microbiome was assessed using Bray-Curtis similarity and was visualised by Principal Component Analysis. Orange Circle-Autoimmune Liver Disease; Green Triangle-Healthy Control; Blue Square-Inflammatory Bowel Disease. Purple Cross-Inflammatory Bowel Disease with Autoimmune Liver Disease.

Microbiome analysis was initially investigated by alpha diversity using both the Chao1 and Shannon diversity indices. Both Chao1 (Figure 1A) and Shannon diversity (Figure 1B) showed similar behaviour with AILD and HC not significantly different, and with IBD-AILD and IBD not significantly different, but with IBD-AILD and IBD both significantly different to both AILD and HC (Table 2 and Table 3). Similar grouping was observed with beta-diversity analysis. AILD and HC were observed to group together with principal component beta-diversity analysis (Figure 2). Similarly, AILD and IBD-AILD are also observed to group together and away from the AILD/HC group (Figure 2). Of interest, four IBD and two IBD-AILD samples grouped together, but away from the main IBD/IBD-AILD group.

### 3.3. Microbiome Analysis—Phyla Distribution

**Figure 3:**
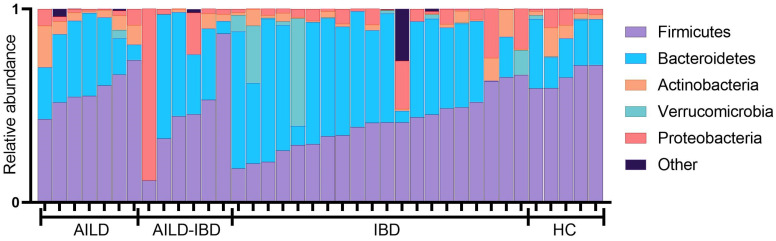
Phyla Distribution. The relative phyla distribution of the predominant intestinal microbiome phyla are presented. AILD (Autoimmune Liver Disease); IBD (Inflammatory Bowel Disease); HC (Healthy Control).

Microbiome composition was further investigated by phyla distribution. Normal phyla distribution, as established by HC samples, showed *Firmicutes* to be the predominant phyla followed by *Bacteroides*, varying amounts of *Actinobacteria* and *Proteobacteria* and minimal *Verrucomicrobia* and other phyla (Figure 3). Similar phyla distribution was observed in AILD participants (Figure 3). Phyla distribution generally differed in AILD-IBD and IBD in that there was greater variability in *Firmicutes* and higher predominance of *Bacteroidetes* compared to HC (Figure 3). Furthermore, some participants in both the IBD and AILD-IBD had divergent phyla composition insofar as *Firmicutes/Bacteroidetes* were the minor phyla with other phyla (including *Proteobacteria*, *Verrucomicrobia* or some combination), being the predominant phyla in the microbiome composition.

## 4. Discussion

The interplay between the gastrointestinal microbiome and the host has been increasingly understood and accepted as a key factor contributing to health and disease states [6]. Dysbiosis and the onset of autoimmune conditions, including inflammatory bowel disease, have been established [4,5]. The link between the intestinal microbiome and autoimmune liver disease, affecting either the parenchyma and/or the biliary system, has, however, been less well-defined. This study has demonstrated that the gastrointestinal microbiome profile of children with AILD resembles that of healthy controls, with no reduction in alpha diversity. The dysbiosis that was found in IBD is similar to that seen in children who have both IBD and AILD.

Microbial communities of the human gastrointestinal tract mainly consist of bacteria from the four predominant phyla of *Firmicutes*, *Bacteroidetes*, *Actinobacteria* and *Proteobacteria* [6]. Although there is substantial variability and debate regarding what constitutes a healthy microbiome, a few general concepts can be observed that are often associated with the microbiome of a host who is free from disease. These concepts include a microbiome with the dominant phyla of *Firmicutes* followed by *Bacteroidetes*, as well as higher diversity [13]. These same concepts can be observed in both the HC and AILD groups of the current study.

Previous work has demonstrated that *Firmicutes* and *Proteobacteria* are increased in AIH and primary biliary cirrhosis in comparison to healthy controls. In PSC, *Bacteroidetes* and *Proteobacteria* are abundant in contrast to *Firmicutes*, which is underrepresented. The AILD group of the current study consisted of four participants with AIH, two with AIH-PSC overlap syndrome and one with seronegative AIH. As there were no participants with PSC in the current cohort, these data were not able to ascertain whether PSC alone may be associated with an altered microbiome profile. Nevertheless, this study does add evidence that, in the absence of IBD, children with AIH and AIH-PSC do not have overtly altered microbiome compared to healthy control children. Furthermore, there was no indication that alpha diversity was decreased in those with AILD, as has been previously reported [14].

There is a relative paucity of work describing the human microbiome in patients with AILD [10]. One study described increased abundance in *Veillonella*, *Klebsiella*, *Streptococcus* and *Lactobacillus* among patients with AILD as compared to healthy controls [15]. A different 2020 study suggested that the microbial signature in patients with AILD were represented by *Lachnospiraceae*, *Veillonella*, *Bacteroides*, *Roseburia* and *Ruminococcaceae* [16]. Taken together, an over-representation of *Veillonella* appears to distinguish patients with AILD from healthy controls [14]. *Bifidobacterium* has been shown to be deplete in AILD, and those with lower *Bifidobacterium* were indeed more likely to not achieve remission of AILD. The lack of difference between AILD and HC in the current study may be a result of the limited sample size and limited microbiome investigations. Nevertheless, the beta diversity analysis indicates that within the current cohort, HC and AILD microbiome composition are essentially similar.

With regards to PSC, microbiome studies have shown an increased prevalence of nine species with a decreased prevalence of another five species. Kummen et al. [7] and Cortez et al. [17] have both demonstrated relative abundance of *Veillonella* genus in young people with PSC. Some data point to a distinction between the microbial signature of patients with PSC-IBD as compared to those with PSC without IBD. This distinction is manifest by an over-representation in *Rothia*, *Lactobacillus*. *Streptococcus* and *Veillonella* in patients with only PSC, whereas *Coprobacillus*, *Eschericia*, *Corneybacterium* and *Lactobacillus* genera appear more related to the coexistence of PSC with IBD. The current cohort was limited to two participants with AIH-PSC, which therefore effectively limited the current study on expanding knowledge of the effect of PSC on the intestinal microbiome.

The implications of better understanding intestinal microbiome in disease states include the discovery of novel therapeutic avenues. Broadly, this could involve modulation of dysbiosis via the provision of prebiotics and/or probiotics, as well as the use of antibiotics for specific indications. There are data to support the use of antibiotics during mild flares of Crohn’s disease [18]. Furthermore, the use of cycling antibiotics and occasionally long-term antibiotics, is known to be beneficial in the context of chronic, or recurrent acute, pouchitis in patients who have undergone colectomy for UC. By the same token, the use of probiotics can be associated with a reduction in the incidence of pouchitis [19]. More recently, there is evidence to support the use of Vancomycin in patients who have PSC, either with or without concomitant IBD [20]. Clearly, one of the risks of more liberal antibiotic usage is the development of multi-resistant organisms. Therefore, any recommendation for the use of anti-microbials in the context of chronic gastrointestinal conditions, which are not due to overt infection, should be ideally supported by robust basic scientific underpinnings.

The current study is limited by the number of patients included and the cross-sectional study design. The strengths of this paper are the comprehensive assessment of the microbiome in each of the study groups, with the inclusion of a group of children with neither liver nor intestinal inflammatory disease. Furthermore, each of the disease groups included children with well-defined disease, reflecting rigorous inclusion and exclusion criteria.

In conclusion, differences in microbiome composition were observed in the AILD-IBD and IBD groups compared to the AILD and HC groups. It has been well established that microbiome profiles in individuals with IBD are different to healthy controls. The observation that AILD clusters with HC and that AILD-IBD clusters with IBD indicated that the altered microbiome in AILD-IBD is likely to be a result of the IBD-associated disease activity, and not the AILD-associated disease activity. Therefore, in this cohort at least, liver disease does not appear to alter the microbiome.

## Figures and Tables

**Table 1:** Characteristics of children included in the evaluation of the intestinal microbiome in setting of Inflammatory Bowel Disease with/without autoimmune liver disease.

	AILD	AILD-IBD	IBD	HC
Number	7	6	22	5
M/F	1M/6F	5M/1F	4M/18F	3M/2F
Age in years: Mean (SD) [Range]	13.3 (3.1) [7.0–16.2]	9.1 (4.2) [11.1–18.0]	11.7 (3.1) [5.4–16.6]	8.1 (5.0) [5.2–16.9]
CD/UC/IBDU/VEO-IBD	NA	2/0/4/0	11/2/7/2	NA
Antibiotics	NA	67%	14%	NA
Steriods	100%	60%	32%	NA
EEN	NA	40%	23%	NA
ASA	NA	60%	77%	NA
MTX	0%	40%	5%	NA
Tacrolimus	14%	20%	9%	NA
Azathioprine	18%	60%	59%	NA
Anti-TNF	0%	20%	5%	NA

IBD = inflammatory bowel disease. AILD = autoimmune liver disease. CD = Crohn disease. UC = ulcerative colitis. IBDU = IBD unclassified. VEO-IBD = very early onset IBD. HC = healthy controls. EEN = exclusive enteral nutrition. ASA= aminosalicylic acid. MTX= methotrexate. TNF = tumour necrosis factor. NA = not applicable. Note: Information was missing for some participants with percentage calculations reflecting the missing information.

**Table 2:** Comparisons between disease groups based in Chao 1 analysis. AILD (Autoimmune Liver Disease); IBD (Inflammatory Bowel Disease); HC (Healthy Control), ns = not significant, **** *p* < 0.0001.

Tukey’s Multiple Comparisons Test	Significant	Summary	Adjusted *p* Value
AILD vs. IBD-AILD	Yes	****	<0.0001
AILD vs. IBD	Yes	****	<0.0001
AILD vs. HC	No	ns	0.9919
IBD-AILD vs. IBD	No	ns	0.9922
IBD-AILD vs. HC	Yes	****	<0.0001
IBD vs. HC	Yes	****	<0.0001

**Table 3:** Comparisons between disease groups based in Shannon’s Diversity. AILD (Autoimmune Liver Disease); IBD (Inflammatory Bowel Disease); HC (Healthy Control), ns = not significant, **** *p* < 0.0001.

Tukey’s Multiple Comparisons Test	Below Threshold?	Summary	Adjusted *p* Value
AILD vs. IBD-AILD	Yes	****	<0.0001
AILD vs. IBD	Yes	****	<0.0001
AILD vs. HC	No	ns	0.9997
IBD-AILD vs. IBD	No	ns	0.9811
IBD-AILD vs. HC	Yes	****	<0.0001
IBD vs. HC	Yes	****	<0.0001

## Data Availability

The data presented in this study are available on request from the corresponding author.

## References

[1] Mieli-Vergani G., Vergani D., Baumann U., Czubkowski P., Debray D., Dezsofi A., Fischler B., Gupte G., Hierro L., Indolfi G. (2018). Diagnosis and Management of Pediatric Autoimmune Liver Disease: ESPGHAN Hepatology Committee Position Statement. J. Pediatr. Gastroenterol. Nutr..

[2] Deneau M.R., El-Matary W., Valentino P.L., Abdou R., Alqoaer K., Amin M., Amir A.Z., Auth M., Bazerbachi F., Broderick A. (2017). The natural history of primary sclerosing cholangitis in 781 children: A multicenter, international collaboration. Hepatology.

[3] Lazaridis K.N., LaRusso N.F. (2016). Primary Sclerosing Cholangitis. N. Engl. J. Med..

[4] Machiels K., Joossens M., Sabino J., De Preter V., Arijs I., Eeckhaut V., Ballet V., Claes K., Van Immerseel F., Verbeke K. (2014). A decrease of the butyrate-producing species Roseburia hominis and Faecalibacterium prausnitzii defines dysbiosis in patients with ulcerative colitis. Gut.

[5] Gevers D., Kugathasan S., Denson L.A., Vázquez-Baeza Y., Van Treuren W., Ren B., Schwager E., Knights D., Song S.J., Yassour M. (2014). The treatment-naive microbiome in new-onset Crohn’s disease. Cell Host Microbe.

[6] Dekaboruah E., Suryavanshi M.V., Chettri D., Verma A.K. (2020). Human microbiome: An academic update on human body site specific surveillance and its possible role. Arch. Microbiol..

[7] Kummen M., Holm K., Anmarkrud J.A., Nygård S., Vesterhus M., Høivik M.L., Trøseid M., Marschall H.U., Schrumpf E., Moum B. (2017). The gut microbial profile in patients with primary sclerosing cholangitis is distinct from patients with ulcerative colitis without biliary disease and healthy controls. Gut.

[8] Sabino J., Vieira-Silva S., Machiels K., Joossens M., Falony G., Ballet V., Ferrante M., Van Assche G., Van der Merwe S., Vermeire S. (2016). Primary sclerosing cholangitis is characterised by intestinal dysbiosis independent from IBD. Gut.

[9] Iwasawa K., Suda W., Tsunoda T., Oikawa-Kawamoto M., Umetsu S., Inui A., Fujisawa T., Morita H., Sogo T., Hattori M. (2017). Characterisation of the faecal microbiota in Japanese patients with paediatric-onset primary sclerosing cholangitis. Gut.

[10] Little R., Wine E., Kamath B.M., Griffiths A.M., Ricciuto A. (2020). Gut microbiome in primary sclerosing cholangitis: A review. World J. Gastroenterol..

[11] Levine A., Koletzko S., Turner D., Escher J.C., Cucchiara S., de Ridder L., Kolho K.L., Veres G., Russell R.K., Paerregaard A. (2014). ESPGHAN revised porto criteria for the diagnosis of inflammatory bowel disease in children and adolescents. J. Pediatr. Gastroenterol. Nutr..

[12] Callahan B.J., McMurdie P.J., Rosen M.J., Han A.W., Johnson A.J., Holmes S.P. (2016). DADA2: High-resolution sample inference from Illumina amplicon data. Nat. Methods.

[13] Ruan W., Engevik M.A., Spinler J.K., Versalovic J. (2020). Healthy Human Gastrointestinal Microbiome: Composition and Function After a Decade of Exploration. Dig. Dis. Sci..

[14] Zheng Y., Ran Y., Zhang H., Wang B., Zhou L. (2021). The Microbiome in Autoimmune Liver Diseases: Metagenomic and Metabolomic Changes. Front. Physiol..

[15] Wei Y., Li Y., Yan L., Sun C., Miao Q., Wang Q., Xiao X., Lian M., Li B., Chen Y. (2020). Alterations of gut microbiome in autoimmune hepatitis. Gut.

[16] Lou J., Jiang Y., Rao B., Li A., Ding S., Yan H., Zhou H., Liu Z., Shi Q., Cui G. (2020). Fecal Microbiomes Distinguish Patients With Autoimmune Hepatitis From Healthy Individuals. Front. Cell. Infect. Microbiol..

[17] Cortez R.V., Moreira L.N., Padilha M., Bibas M.D., Toma R.K., Porta G., Taddei C.R. (2020). Gut Microbiome of Children and Adolescents With Primary Sclerosing Cholangitis in Association With Ulcerative Colitis. Front. Immunol..

[18] Su J.W., Ma J.J., Zhang H.J. (2015). Use of antibiotics in patients with Crohn’s disease: A systematic review and meta-analysis. J. Dig. Dis..

[19] Gionchetti P., Calabrese C., Lauri A., Rizzello F. (2015). The therapeutic potential of antibiotics and probiotics in the treatment of pouchitis. Expert Rev. Gastroenterol. Hepatol..

[20] Tabibian J.H., Weeding E., Jorgensen R.A., Petz J.L., Keach J.C., Talwalkar J.A., Lindor K.D. (2013). Randomised clinical trial: Vancomycin or metronidazole in patients with primary sclerosing cholangitis—A pilot study. Aliment. Pharmacol. Ther..

